# Positional differences in peak- and accumulated-training load relative to match load in highly-trained female football players

**DOI:** 10.3389/fspor.2026.1799448

**Published:** 2026-04-01

**Authors:** Ivan Baptista, Pedro Figueiredo, Dag Johansen, Svein Arne Pettersen

**Affiliations:** 1 Department of Computer Science, Faculty of Science and Technology, UiT The Arctic University of Norway, Tromsø, Norway; 2 Physical Education Department, College of Education, United Arab Emirates University, Al Ain, United Arab Emirates; 3 Research Center in Sports Sciences, Health Sciences and Human Development, CIDESD, Maia, Portugal; 4 School of Sport Sciences, Faculty of Health Sciences, UiT The Arctic University of Norway, Tromsø, Norway

**Keywords:** external load, high-speed distance, microcycle, playing position, women's football

## Abstract

**Introduction:**

The evaluation of the physical load of the training process is paramount not only as an attempt to decrease injury rates or to verify the effectiveness of interventions but mostly to find the best way to stimulate the players and optimise their physical capacity. Literature has focused mainly on professional meńs football and future research on female football is mandatory. Thus, this study aimed to compare a) the peak periods in training sessions and matches and b) the accumulated-training load of microcycles and official matches by playing position in female football players.

**Methods:**

A total of 80 female football players competing in the Norwegian top-division were involved in this study in which match load (ML) and training load (TL) from a total of 103 official matches and 120 training sessions were collected. Physical parameters analysed included total distance (TD), acceleration distance (Acc_dist_), high-speed running distance (HSRD), sprint distance (SpD), 5-min peak TD (TD_peak_), 30-sec peak of HSRD (HSRD_peak_), 30-sec peak of SpD (SpD_peak_), and maximum speed.

**Results:**

While TD covered during the microcycle was higher than in the match for every playing position, no significant differences were observed for HSRD and SpD. While TD_peak_ in match was higher for FB, CM, WM and FW (*p* < 0.001), differences in HSRD_peak_ were only observed for FB (*p* < 0.001) and WM (*p* = 0.002).

**Discussion:**

The present study corroborates the evidence reported in men's football, where the lower training/match ratios of HSRD and SpD when compared with TD and accdist reveal that the common build-up of training weeks might not give the contextual opportunities for the appearance and/or accumulation of high-speed actions.

## 1 Introduction

In football, monitoring training and match load has become a daily practice. This load quantification is indispensable for planning, structuring, evaluating, and optimising the training process (1). Despite being well documented (2–4) and representing the greatest physiological stimulus of the week, the match is only responsible for ∼20% of the weekly load (5). Thus, the evaluation of the physical load of the training process is paramount not only as an attempt to decrease injury rates or to verify the effectiveness of interventions (6, 7) but mostly to find the best way to stimulate the players and optimise their physical capacity (8, 9). A recent systematic review (5) of the monitoring of training and match load reported only one study conducted on female players (10), concluding that the literature has focused mainly on professional men's football and that future research on female football is mandatory.

Due to the continuous and exponential increase of participation rates during the past decade, the increased investment in the development of players and coaches, and the consequent professionalisation of women's football (11), it is expected that the demands and dynamics of training and match events have evolved (12). There is sparse information and scientific literature on match demands and even less connecting match load to training load in women's football (13, 14).

Moreover, recent research suggests that increased professionalisation of the sport is related to the observed increases in physical performances of female elite players during match-play (15). In addition, evidence-based practices in women's football should not be supported by reference values for men's football due to the apparent differences in anthropometrics, strength, and fitness status (8, 16), as well as the greater performance variability observed in female compared to male athletes (17–19).

Besides that, more important than knowing the total match/training demands is to take the stochastic nature of the football game into account (20) by analysing the pattern in which such activity is performed and understanding what the implications of unequal distributions of match/training loads are. Indeed, research within the match analysis domain is no longer limited to reporting mean values, and practitioners are now more conscious that preparation for the most intense periods of match-play (peak periods) is fundamental to reaching a high-performance level (21). Harkness-Armstrong et al. (22) concluded in their systematic review of women's football research that discrete periods likely underestimate peak periods by up to 25% compared to moving average analysis and that relative intensities decrease as the duration of the epoch length increases. Thus, moving averages and short epoch lengths (<5 min) are strongly recommended when analysing peak periods, particularly for high-speed metrics (23).

Another recent systematic review of the peak periods in professional football matches (24) included only two studies (out of 12) using women's professional teams (25, 26). On a broader review of the match characteristics of women's football across different levels (22), the authors considered eight studies reporting peak periods. However, only half of the results were discriminated by playing position, only two adopted the moving average method, and only one study (27) used epoch lengths shorter than 5 min.

Whilst the nomenclatures used to define playing positions are challenging to uniformise, high levels of categorisation (i.e., defenders, midfielders and attackers) should be avoided since significant differences exist between central and wide positions (27, 28). According to Harkness-Armstrong et al. (22), this challenge is particularly problematic in studies available with female football teams, with only nine of the twenty-six studies included in their review differentiated central and wide positions of defenders and midfielders. Baptista et al. (29) analysed both peak- and accumulated-training load relative to match load in professional male football players, and the differences observed across playing positions reveal a lack of position specificity in the training drills/sessions implemented even at top level.

Research with women has recently been published (9, 30), yet possible methodological issues and bias may arise since these authors have conducted single-club studies and used relatively small sample sizes (22 and 37 players, respectively). Moreover, while the study of Olaizola et al. (9) has not investigated the peak periods, Savolainen et al. (30) compared these periods between match-play and different types of sided games. However, the whole training process encompasses a broader range of training methods, such as position-specific drills, individualized running-based, and different football-specific exercises that must be considered when quantifying training demands to avoid underestimating overall training stimulus.

Therefore, this study aimed to quantify and compare a) the peak periods in training sessions and matches and b) the accumulated-training load of typical microcycles (7-day microcycles) and official matches by playing position. We hypothesise that high-speed variables will present significantly lower training/match ratios when compared with total distance or acceleration demands, and that the most physically demanding playing positions in the match will present lower training/match ratios than less demanding positions.

## 2 Methods

### 2.1 Subjects

Before initiating the study, we sought ethical approval from the Regional Committee for Medical and Health Research Ethics-Northern Norway (reference number 53884). We were exempted as the data collection did not include a biobank, medical, or health data related to illness, nor interfered with the regular operation of the players. Approval from the Norwegian Centre for Research Data (reference number: 296155) and written informed consent from 80 female football players (22.3 ± 3.7 years of age) representing four teams in the Norwegian premier division were obtained. The players involved in the present study were classified as highly trained according to the criteria outlined by McKay et al. (31). Goalkeepers were not included in this study, and players were divided into five different playing positions: centre-backs (CB) (*n* = 15; match observations M_obs_ = 157; training observations T_obs_ = 241), fullbacks (FB) (*n* = 15; M_obs_ = 105; T_obs_ = 199), centre midfielders (CM) (*n* = 32; M_obs_ = 212; T_obs_ = 502), wide midfielders (WM) (*n* = 7; M_obs_ = 29; T_obs_ = 93), and centre forwards (FW) (*n* = 11; M_obs_ = 34; T_obs_ = 122). These positions were chosen according to the teams’ most used tactical formations and previous research (3).

### 2.2 Procedures and data collection

From March 2020, we conducted a prospective observational study in which match load (ML) and training load (TL) from a total of 103 official matches (M_obs_ = 537) and 120 training sessions (T_obs_ = 1,157), spanning two entire seasons, were collected using STATSports Apex (Newry, Northern Ireland), with a sampling frequency of 10 Hz. The tracking system's validity and accuracy (bias=1%–2%) have been presented elsewhere (32). All training sessions and home matches were played on artificial grass, with only occasional away matches on natural grass. Players wore their Global Positioning System (GPS) unit on their upper back during matches, adhering to manufacturer instructions. To minimize inter-device error, each player consistently used the same GPS unit throughout data collection (32).

Match activity profiles (excluding the warm-up) per playing position were characterised. Match data was analysed if: a) players completed the entire match, and b) the player played in only one position during the respective match. T_obs_ from players without M_obs_, and from players who did not complete the training session were excluded from the analysis. The teams recruited for this study were involved in two domestic competitions each (league and cup) and played only one official match per week. External load data of 32 typical 7-days microcycles was considered for analysis. The microcycles analysed included one day-off, five training sessions, and the match-day. Eventual compensatory or recovery sessions were excluded, and only full-team sessions were considered, composed by warm-up exercises, a combination of small- to large-sided games, and a plethora of tactical-technical drills.

### 2.3 Data analysis

Physical parameters analysed included total distance (TD), acceleration distance (Acc_dist_), high-speed running distance (HSRD), sprint distance (SpD), 5-min peak TD (TD_peak_), 30-sec peak of HSRD (HSRD_peak_), 30-sec peak of SpD (SpD_peak_), and maximum speed. The “peak” variables refer to the individualized values on the 30-sec or 5-min period of the match where those variables presented the highest scores. These peak periods were identified by applying a 30-sec or 5-min rolling average to each metric. The peak period duration was chosen according to previous research in women's football (23), where the authors suggested the use of short epoch lengths (15–30 s) for the analysis of intensity-related variables and longer periods (5-min) for volume-related metrics. The cumulative load per variable was obtained by summing the values of the five training sessions of each microcycle. Speed thresholds for HSRD (>16 km/h) and SpD (>20 km/h) were defined according to the suggestion of (33) for research involving female athletes. In addition, Acc_dist_ was defined as the distance covered with a positive or negative change in speed of more than ±2.26 m/s^−2^, finishing when the rate of acceleration/deceleration reached 0 m/s^−2^.

### 2.4 Handling of missing data

To handle missing data, we followed recommendations by Bache-Mathiesen et al. (34), Borg et al. (35), and Malone et al. (36). First, we set all physical performance variables as missing on sessions with a mean horizontal dilution of precision >5 or a mean number of satellites <12. We also set peak speed as missing if above 32 km/h based on theoretical max speed values of 29.2 ± 1.4 km/h in a similar cohort (37).

### 2.5 Statistical analysis

TD, Acc_dist_, maximum speed, TD_peak_, HSRD_peak_, and SpD_peak_ were modelled in R using the lme4 package (38), with REML estimation, while HSRD and SpD were modelled in the same software using glmmTMB (39). TD, Acc_dist_, HSRD, and SpD models included the interaction between day and playing position (CB, CM, FB, FW, WM) as fixed effects, while models for maximum speed, TD_peak_, HSRD_peak_, and SpD_peak_ included the interaction between type of day (match vs. training) and playing position as fixed effects. In addition, HSRD and SpD were modelled using the Tweedie family with a log link function and dispersion heterogeneity was modeled as a function of day to accommodate variance differences.

Random-effects structure was specified for TD, HSRD and SpD with Player-specific random intercepts and random slopes for match vs. training day, team-specific random intercepts and random slopes and week-level random intercepts for time-based temporal trends. For Acc_dist_, team random slopes were excluded due to convergence issues, retaining team random intercepts and player slopes and week effects. For maximum speed, TD_peak_, HSRD_peak_, and SpD_peak_ random structure included player-specific random intercepts and random slopes for match vs. training day and a team-specific random intercept.

Model selection was done via Akaike Information Criterion, Bayesian Information Criterion, and likelihood ratio tests. Model diagnostics were performed using DHARMa simulation-based tests (*n* = 1,000 simulations) (40) for Tweedie models assessing uniformity, dispersion, zero-inflation, and outlier frequency, and performance package checks (41) for Gaussian models evaluating residuals normality and homoscedasticity, and influential observations, supplemented by visual diagnostics (residual plots, QQ plots).

The differences in load measures between training (accumulated) and match were examined by position. Additionally, for the peak analysis, we examined, per position, the differences in peaks between the training (maximum values) and match conditions. For these comparisons, specific contrasts were developed, and the emmeans package (42) was used to compute estimated marginal means and response ratios, employing the multivariate t-distribution adjustment method to account for multiple comparisons. In addition, for the peak analysis, *p*-values were estimated using permutation tests (*n* = 1,000 permutations), in which the match vs. training labels were randomly shuffled. Model-level effect sizes were quantified using marginal and conditional R^2^ for Gaussian models following Nakagawa et al. (43) using the performance package (41). For Tweedie models (HSRD, SpD), likelihood-ratio R^2^ (Cox-Snell) was computed using MuMIn (44).

The α-level was set at 0.05 as the level of significance. Unless otherwise stated, all results are reported as estimated marginal means or response ratios and 95% confidence interval.

For accumulated load models, TD showed marginal R^2^ = 0.823 and conditional R^2^ = 0.864, and Acc_dist_ showed marginal R^2^ = 0.644 and conditional R^2^ = 0.766. For Tweedie models, likelihood-ratio R^2^ (Cox-Snell) was 0.330 for HSRD and 0.264 for SpD. For peak models, MaxSpeed showed marginal R^2^ = 0.164 and conditional R^2^ = 0.458; TD_peak_ showed marginal R^2^ = 0.295 and conditional R^2^ = 0.487; HSRD_peak_ showed marginal R^2^ = 0.097 and conditional R^2^ = 0.244; and SpD_peak_ showed marginal R^2^ = 0.127 and conditional R^2^ = 0.281.

## 3 Results

### 3.1 Accumulated training load

**Figure 1:**
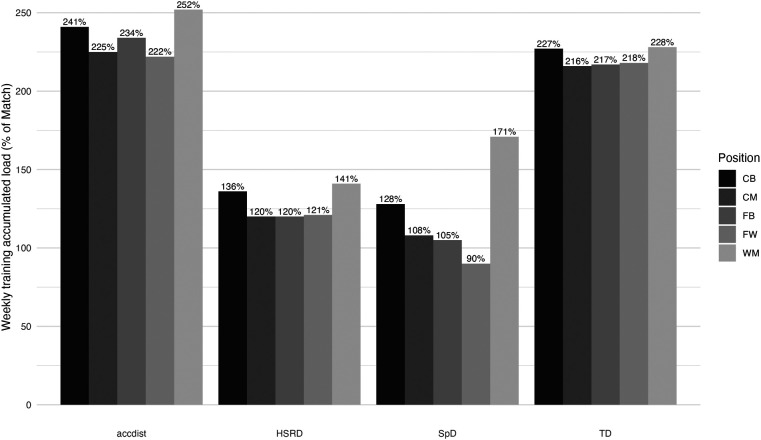
Microcycle accumulated-training load in percentage of match load.

Table 1 presents the comparison between the absolute match-day values and the accumulated training load of typical microcycles for different metrics, across playing position. For every variable analysed, all playing positions accumulated higher distances during microcycles than in match, with exception for SpD of FW [260.3 (156.9, 363.8) vs. 288.4 (199.9, 377.0)], where distances covered were higher during matches. While TD covered during the microcycle was significantly higher than in the match for every playing position, no significant differences were observed in HSRD and SpD for any playing position. A clear pattern is possible to identify in Figure 1, with all playing positions largely overperforming match demands during training microcycles for TD (range: 216%–228%) and Acc_dist_ (range: 222%–252%), while lower percentages were observed for HSRD (range: 120%–141%) and SpD (range: 90%–171%).

**Table 1:** Estimated marginal mean [95% confidence interval] of absolute match values, accumulated training load, grouped by playing position, with match-day vs. training differences with associated p-value and their corresponding response ratio [95% confidence interval].

Playing position	Metric	Mean [95% confidence interval]		Difference (MD-training)	*P*-value	Ratio [95% confidence interval]
		Match-day	Training microcycle			
CB	TD	9,248.4 [8,872.8, 9,624.1]	20,975.4 [17,775.0, 24,175.7]	−11,726.9 [−14,793.2, −8,660.7]	<0.001	2.27 [1.94, 2.65]
	Acc_dist_	1,493.8 [1,255.2, 1,732.3]	3,597.8 [2,543.9, 4,651.7]	−2,104.1 [−2,976.4, −1,231.7]	0.004	2.41 [1.95, 2.97]
	HSRD	675.3 [579.1, 771.5]	921.6 [669.7, 1,173.4]	−246.3 [−481.3, −11.2]	0.118	1.36 [1.05, 1.77]
	SpD	181.3 [136.8, 225.8]	232.0 [149.2, 314.8]	−50.7 [−129.4, 27.9]	0.508	1.28 [0.90, 1.82]
FB	TD	10,074.8 [9,671.2, 10,478.5]	21,888.2 [18,663.8, 25,112.6]	−11,813.4 [−14,914.6, −8,712.2]	<0.001	2.17 [1.87, 2.52]
	Acc_dist_	1,837.8 [1,595.9, 2,079.7]	4,301.5 [3,244.3, 5,358.7]	−2,463.7 [−3,348.1, −1,579.4]	0.001	2.34 [1.95, 2.81]
	HSRD	1,045.3 [892.9, 1,197.8]	1,256.1 [903.4, 1,608.8]	−210.7 [−542.0, −120.5]	0.487	1.20 [0.92, 1.57]
	SpD	305.1 [228.1, 382.1]	321.5 [203.3, 439.7]	−16.4 [−131.8, 98.9]	0.998	1.05 [0.73, 1.51]
CM	TD	10,442.9 [10,080.4, 10,805.4]	22,552.0 [19,213.5, 25,890.5]	−12,109.1 [−15,314.8, −8,903.5]	0.001	2.16 [1.86, 2.51]
	Acc_dist_	1,892.4 [1,668.4, 2,116.5]	4,254.5 [3,163.1, 5,345.9]	−2,362.1 [−3,255.1, −1,469.1]	0.006	2.25 [1.89, 2.67]
	HSRD	1,028.8 [916.3, 1,141.4]	1,238.1 [923.4, 1,552.8]	−209.3 [−509.4, 90.9]	0.411	1.20 [0.94, 1.54]
	SpD	269.5 [219.9, 319.1]	290.2 [197.0, 383.4]	20.7 [−116.1, 74.76]	0.984	1.08 [0.77, 1.50]
WM	TD	10,234.9 [9,566.9, 10,903.0]	23,302.3 [19,274.9, 27,329.6]	−13,067.3 [−17,011.9, −9,122.8]	<0.001	2.28 [1.92, 2.69]
	Acc_dist_	1,849.3 [1,533.8, 2,164.8]	4,666.9 [3,414.9, 5,918.8]	−2,817.6 [−3,930.2, −1,704.9]	<0.001	2.52 [2.01, 3.17]
	HSRD	1,156.7 [893.3, 1,420.0]	1,627.3 [1,094.3, 2,160.3]	−470.6 [−965.5, 24.2]	0.174	1.41 [1.02, 1.93]
	SpD	267.1 [153.9, 380.3]	456.7 [239.2, 674.2]	−189.6 [−382.1, 2.9]	0.164	1.71 [1.07, 2.73]
FW	TD	9,882.6 [9,363.3, 10,401.8]	21,563.7 [18,414.7, 24,712.6]	−11,681.1 [−14,734.5, −8,627.7]	<0.001	2.18 [1.86, 2.55]
	Acc_dist_	1,855.2 [1,585.2, 2,125.2]	4,113.4 [3,073.7, 5,153.2]	−2,258.3 [−3,141.4, −1,375.2]	0.001	2.22 [1.80,2.74]
	HSRD	1,031.2 [849.8, 1,212.5]	1,247.3 [885.1, 1,609.6]	−216.2 [−561.7, 129.3]	0.501	1.21 [0.91, 1.61]
	SpD	288.4 [199.9, 377.0]	260.3 [156.9, 363.8]	28.1 [−81.2, 137.4]	0.967	0.90 [0.60, 1.35]

TD, Acc_dist_, HSRD and SpD values presented in meters.

### 3.2 Peak periods

**Figure 2:**
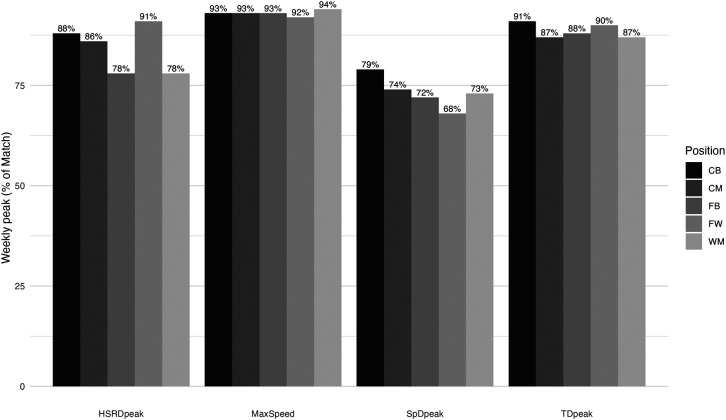
Microcycle training peak in percentage of match peak.

Significant differences between matches and training microcycle were observed in different metrics for every playing position (Table 2). However, in contrast with the absolute and accumulated-training load, the peak periods were always higher during match than throughout the training microcycle. While TD_peak_ in match was significantly higher for every playing position (except for CB) than in trainings (*p* < 0.001), significant differences in HSRD_peak_ were only observed for FB (*p* < 0.001) and WM (*p* = 0.002). Moreover, when analysing training peaks as a percentage of match peaks (Figure 2), it is possible to identify that TD_peak_ is the most stable variable between playing positions (range: 87%–91%), while considerably larger differences are observed in HSRD_peak_ (78%–91%) and SpD_peak_ (68%–79%).

**Table 2:** Estimated marginal mean [95% confidence interval] of peak periods and maximum speed, grouped by playing position, with match-day vs. training differences with associated p-value and their corresponding response ratio [95% confidence interval].

Playing position	Metric	Mean [95% confidence interval]		Difference (MD-training)	*P*-value	Ratio [95% confidence interval]
		Match-day	Training microcycle			
CB	TD_peak_	622.6 [600.2, 645.1]	568.6 [538.1, 599.0]	54.1 [29.2, 79.0]	0.014	0.91 [0.87, 0.96]
	HSRD_peak_	55.5 [51.0, 60.0]	48.7 [42.9, 54.6]	6.7 [−0.3, 13.7]	0.309	0.88 [0.76, 1.01]
	SpD_peak_	33.9 [29.3, 38.5]	26.9 [22.0, 31.8]	7.0 [1.6, 12.4]	0.130	0.79 [0.66, 0.96]
	MaxSpeed	25.9 [25.0, 26.8]	24.2 [23.3, 25.0]	1.7 [1.0, 2.4]	0.002	0.93 [0.91, 0.96]
FB	TD_peak_	679.1 [655.5, 702.8]	599.3 [568.1, 630.4]	79.9 [53.3, 106.4]	<0.001	0.88 [0.84, 0.92]
	HSRD_peak_	68.0 [63.1, 73.0]	52.9 [46.8, 59.1]	15.1 [7.6, 22.6]	<0.001	0.78 [0.68, 0.89]
	SpD_peak_	43.9 [38.9, 48.8]	31.4 [26.3, 36.5]	12.5 [6.7, 18.3]	0.002	0.72 [0.60, 0.85]
	MaxSpeed	26.1 [25.2, 27.0]	24.3 [23.4, 25.2]	1.8 [1.0, 2.5]	<0.001	0.93 [0.90, 0.96]
CM	TD_peak_	697.9 [676.5, 719.3]	604.7 [580.7, 628.7]	93.2 [75.1, 111.2]	<0.001	0.87 [0.84, 0.90]
	HSRD_peak_	61.0 [57.4, 64.6]	52.5 [48.3, 56.6]	8.5 [3.5, 13.6]	0.133	0.86 [0.78, 0.95]
	SpD_peak_	38.4 [34.4, 42.5]	28.6 [24.6, 32.6]	9.8 [5.9, 13.8]	0.010	0.74 [0.65, 0.85]
	MaxSpeed	25.9 [25.1, 26.7]	24.1 [23.3, 24.9]	1.8 [1.3, 2.3]	<0.001	0.93 [0.91, 0.95]
WM	TD_peak_	709.3 [672.3, 746.1]	619.4 [576.6, 662.2]	89.9 [49.1, 130.6]	<0.001	0.87 [0.82, 0.93]
	HSRD_peak_	61.66 [52.5, 70.8]	48.3 [39.6, 57.0]	13.4 [1.8, 24.9]	0.002	0.78 [0.63, 0.97]
	SpD_peak_	39.4 [31.0, 47.8]	28.9 [21.9, 35.9]	10.5 [1.3, 19.6]	0.008	0.73 [0.56, 0.96]
	MaxSpeed	26.0 [24.7, 27.4]	24.5 [23.4, 25.7]	1.5 [0.3, 2.7]	0.008	0.94 [0.90, 0.99]
FW	TD_peak_	667.8 [638.2, 697.4]	601.6 [566.2, 637.0]	66.2 [31.0, 101.4]	0.001	0.90 [0.85, 0.95]
	HSRD_peak_	56.0 [48.8, 63.2]	51.0 [43.8, 58.1]	5.0 [−4.8, 14.8]	0.586	0.91 [0.76, 1.10]
	SpD_peak_	38.3 [31.7, 44.9]	25.8 [20.0, 31.6]	12.4 [4.7, 20.2]	0.002	0.68 [0.52, 0.87]
	MaxSpeed	26.4 [25.3, 27.5]	24.2 [23.2, 25.1]	2.2 [1.2, 3.2]	<0.001	0.92 [0.88, 0.95]

TD_peak_, HSRD_peak_ and SpD_peak_ values presented in meters. MaxSpeed presented in km/h.

## 4 Discussion

In this study, we objectively quantified and compared, per playing position, the peak periods and the accumulated-training load with match demands. Consistent with our hypothesis, when analysing the accumulated-training load, high-speed variables (HSRD and SpD) presented considerably lower training/match ratios than TD and Acc_dist_. Although all playing positions overperformed match demands during training microcycles for every metric analysed, significant differences were only observed in TD (CB, FB, CM, WM, and FW) and Acc_dist_ (CB, FB, WM and FW).

Monitoring the training load is fundamental to provide practitioners with a clear idea of how the weekly stimuli differed from match demands (45). To facilitate training load interpretations, the communication between practitioners, and training prescription, previous research (29, 46) has used match load as a reference. We followed that approach in Figures 1 and 2 by using the cumulative load, 5-min peaks (TD), and 30-sec peaks (HSRD and SpD), expressed as a percentage of match values (100%).

Figure 1 shows that the accumulated distance covered at the most demanding speed thresholds (HSRD and SpD) during training weeks was always closer to full-match values than TD and Acc_dist_, which largely exceeded the usual demands of official matches. This is partially corroborated by previous research with Norwegian (29), Dutch (46), and Portuguese (45) male teams, where acceleration metrics were largely overperformed during training weeks in contrast to high-speed metrics. These findings support the idea that current training methodologies used in women's football also emphasize acceleration demands compared to high-speed metrics. This could be caused by the typology of drills used, such as small-sided games, where the prevalence of small areas may lead to a decrease in opportunities for the players to reach high speed and consequently to an increase in the frequency of accelerations (47).

While TD values observed align with the theoretical targets suggested in the literature for endurance training (2.0–2.5 times the competition distance as total weekly training volume), evidence-based recommendations on other metrics do not currently exist (48). The results observed in this study are extremely important as practitioners must be aware that alternating the speed across short distances (acceleration) is more metabolically demanding than constant velocity movements. In addition, higher mechanical stress caused by eccentric muscle contraction is associated with exposure to decelerations during small-sided games (49). Moreover, the higher variability of high-speed metrics observed in training and matches (17, 50) results from the unpredictable nature of match-play and game-based drills. Thus, practitioners are advised to complement these types of training drills with other forms of training (e.g., running-based drills).

However, while the study of Baptista et al. (29) reported very low accumulated-training load for every playing position in SpD (range: 36%–61%) and HSRD (range: 57%–71%), in the present study, these discrepancies between accumulated weekly load and match demands in high-speed metrics were not evident. Besides that, only FW (90%) accumulated lower SpD during the training week than in the match, with HSRD being overperformed in training by every playing position. It is important to note that the higher accumulated-training load in high-speed metrics during the microcycle in this study, compared to previous research, might be influenced by the lower cut-off values applied for HSRD (16 km/h) and SpD (20 km/h), which naturally increase the volume of high-speed running captured, and were specifically adapted for women's football.

The peak period analysis, shown in Figure 2, reveals that regardless of the metric used, peak period intensity during training sessions was not as demanding as the peak periods experienced during match-play. Despite the most intense periods of match play being often linked to goals or goal-scoring opportunities (51), the applicability of peak match demands as benchmarks for training sessions is still controversial (17). A similar trend was observed with MaxSpeed, where the highest values appear in match-play, with the players achieving 92%–94% of their maximum speed during training microcycles. This maximum or near-to-maximum speed exposure somewhat aligns with the guidelines of speed exposure available in the literature, as practitioners are advised to expose the players to 1–2 runs >90%–95% maximal sprinting speed (52). This is crucial to prepare the players for the match demands properly and to reduce the potential injury risk (53).

### 4.1 Limitations and further research

Even though this is the first large-scale study to compare training and match load data in women's football, some limitations should be acknowledged. Despite the undoubtful added value of using individualized thresholds for external load analysis, only standardized thresholds were used in this study. Absolute speed cut-offs can misclassify high-speed running and sprinting demands in women's football because they do not account for large individual differences in maximal velocity, leading to under- or overestimation of actual intensity. Using individual, percentage-based thresholds provides a more accurate representation of each player's physical load relative to their own capacity, improving both monitoring and training prescription.

In addition, methodological decisions on inclusion and exclusion criteria, together with the fact that nowadays, players of specific playing positions tend to be substituted more often than others, resulted in some positional subgroups having a sample size three to four times smaller than others. Furthermore, due to methodological limitations, we only analysed univariate peak periods, so different conclusions could be made if multivariate peak periods were considered. The general training week analysis might not provide specific enough insights into the locomotor demands of each training drill used. Future research should aim to improve the characterization of the locomotor and technical characteristics of different training drills to better assist the planning and design of training sessions. Moreover, because the dataset spans two seasons and multiple teams, contextual factors such as season phase, playing surface, match location, competitive context, number of training sessions and microcycle duration may have influenced both accumulated-training load and peak match demands. These sources of variability limit the generalisability of the proposed training benchmarks when applied to other leagues, competition levels, or environments with different contextual constrains. Finally, a very limited understanding remains regarding the match and training demands of women's football goalkeepers, and the present study has not improved that field.

## 5 Conclusions

This study provides novel insights into the positional differences in peak and accumulated training load relative to match load in elite female football players. Our findings corroborate the evidence already observed in men's football, where the considerably lower training/match rations of HSRD and SpD when compared with TD and Acc_dist_ reveal that the common build-up of training weeks might not give the contextual opportunities for the appearance and/or accumulation of high-speed actions. However, such a challenge does not seem to exist regarding accelerations, with Acc_dist_ during the microcycle largely exceeding the match demands. These results highlight the importance of position-specific training adjustments and offer foundational data for establishing evidence-based training targets in women's football, contributing to optimized player's performance development and injury prevention strategies. This study may also be used by researchers and practitioners as a starting point for the establishment of theoretical targets for high-speed metrics, as it already exists for other metrics such as TD and accelerations.

## Data Availability

The raw data supporting the conclusions of this article will be made available by the authors, without undue reservation.
